# To what extent the weight changes impact the risk of hypertension among menopausal women: insights from Tehran lipid and glucose study

**DOI:** 10.1186/s12905-024-02974-8

**Published:** 2024-02-19

**Authors:** Marzieh Saei Ghare Naz, Maryam Mousavi, Mahsa Noroozzadeh, Maryam Farahmand, Fereidoun Azizi, Fahimeh Ramezani Tehrani

**Affiliations:** 1 Reproductive Endocrinology Research Center, Research Institute for Endocrine Sciences, Shahid Beheshti University of Medical Sciences, Tehran, Iran; 2 Endocrine Research Center, Research Institute for Endocrine Sciences, Shahid Beheshti University of Medical Sciences, Tehran, Iran; 3 The Foundation for Research & Education Excellence, AL, USA

**Keywords:** Metabolic syndrome, Weight, Menopause, Hypertension, Obesity, Weight loss

## Abstract

**Background & aim:**

The association between weight change and incident hypertension (HTN) in menopausal women has not been well characterized. This study aimed to determine whether weight changes after menopausal years make a difference in incidents of hypertension.

**Materials & methods:**

This population-based study was performed using data collected from Tehran Lipid and Glucose Study cohort (1999–2018). Women who had natural and early menopause were followed up every 3 years. Data gathering was performed through the standard protocol of the study. Statistical analysis was performed using multivariable Cox hazard regression analysis. We used the ‘survival’ package in the R software version 3.6.0 to fit survival models.

**Results:**

A total of 487 menopausal women met the inclusion criteria; 62.6% had natural menopause and remained had early menopause. Among the participants, 65.5% experienced HTN. The highest proportion of participants had > 5% weight gain, while the lowest had 3–5% weight gain. Either losing body weight (lost > 5%: HR: 0.44; CI 95%, 0.32, 0.62; *p* < 0.001), (lost 3–5%; HR: 0.47; CI 95%, 0.26, 0.84; *p* = 0.01), and weight gain > 5% (HR: 0.69; CI 95%, 0.51, 0.91; *p* = 0.01), were associated with decreased risk of HTN after adjustment for confounders. In this study, weight loss and gain have a protective impact on the development of HTN in subjects. For incident HTN, age (HR: 1.04 (1.01, 1.08), *p* = 0.004), fasting blood glucose (HR: 1.01, CI 95%:1.00, 1.01; *p* < 0.001), body mass index (1.02 (1.00, 1.05), *p* = 0.03) and smoking (1.70 (1.11, 2.58), *p* = 0.01) were positively associated with HTN.

**Conclusions:**

Our study indicates the significant association of weight change with hypertension risk in later life among menopausal women.

**Supplementary Information:**

The online version contains supplementary material available at 10.1186/s12905-024-02974-8.

## Introduction

Menopause, a permanent cessation of menses for at least 12 months, is a turning point in female health which can greatly impact their cardio-metabolic health [1]. Menopause occurs due to pathologic and non-pathologic conditions. Natural transition of menopause occurs on average at 51 years old, while estrogen deficiency due to pathological conditions could result in early (40–45 years) or late menopause (> 55 years) [2]. Menopausal timing is greatly influenced by genetic and non-genetic factors such as socio-demographic differences, reproductive history, body mass and composition and various environmental factors [3]. Menopause is one of the leading timing points of weight gain in every woman’s life. It is said that the average weight gain during menopause is 2–5 pounds [4]. However, according to the menopause society recommendations, the average weight gain is 2.3 kg, and this alteration is mainly due to ageing and lifestyle changes than menopause itself [5]. Age at the onset of natural menopause and menopausal symptoms can profoundly affect the female’s risk for cardio-metabolic abnormality [1].

Changes in body fat distribution from a gynoid to an android pattern, metabolic alteration, endothelial dysfunction and vascular inflammation can contribute to an increased risk of cardiovascular disease (CVD) in menopausal women [6]. Additionally, the menopause transition can accelerate the cardiovascular disease risk [7, 8]. It is proposed that chronological ageing has a key role in weight change during menopause, while ovarian ageing is mainly associated with altering body composition and fat distribution [9]. In other words, hormonal change during menopause is associated with total body and abdominal fat alteration [10]. Changes in body weight are coincident with changes in cardio-metabolic disorders risk factors. Indeed weight changes act as a double-edged sword; unintentional weight loss and weight gain predict the increased risk for CVD [11]. During the menopausal period, structural and functional changes can predict the development of hypertension (HTN) [12]. Rurik et al., in a retrospective study, found that weight gain in menopausal women is a significant risk factor for incident diabetes and HTN [13]. It has recently become evident that obesity and HTN have rapidly risen worldwide [14, 15]. HTN, obesity and being overweight are more prevalent among postmenopausal women [16]. Hypertension is defined by the European Society of Cardiology and European Society of Hypertension (ESC/ESH) guidelines as blood pressure values higher than 140/90 mmHg [17]. Prevalence of HTN among a sample of Iranian postmenopausal women is about 50% [18]. And the direct costs of HTN treatment in Iran were 87.54 million USD [19]. In different regions of the world, between 30 and 75% of postmenopausal women are hypertensive [20, 21]. Factors contributing to developing HTN in postmenopausal women include genetic factors, environmental factors, and alteration in sex hormone levels, however, the main relative contributors of HTN after menopause are not completely known and there are conflicting findings [22].

HTN can pose negative effects on sexual function and quality of life of menopausal women [23, 24]. Diagnosis and treatment of HTN in postmenopausal women are suboptimal, which can increase the burden of CVD and its related complications [20]. The role of treatments and lifestyle changes on blood pressure in postmenopausal women was discussed in previous studies. A recent trial supported that combined exercise training among postmenopausal women can lead to great reduction of blood pressure (BP) [25]. Furthermore, a meta-analysis reported that postmenopausal women with resistance training without hormone therapy had lower blood pressure than those in the control group [26]. A randomized, parallel dietary intervention study showed that Low-sodium Dietary Approaches to Stop Hypertension (DASH) diets can lead to reducing BP among postmenopausal women [27].

Obesity in postmenopausal women may be attributed to the dysregulation of lipid metabolism and reduced energy expenditure following decreasing physical activity [28]. This is also associated with predominant abdominal fat accumulation [29]. Cardio-metabolic abnormalities are likely to be escalated by biological alterations that occur through menopause. Recent review well-discussed the protective effect of estrogens on cardio-metabolic health, which has been broadly associated with reducing oxidative stress and fibrosis and improving mitochondrial function [30]. Despite the existence of studies which revealed the excess risk of HTN after menopause, the impact of weight change in this period is not clear. It is unknown how weight change during menopause may affect the development of HTN in women. This study aimed to determine whether the weight change among menopausal women impacts the risk of HTN.

## Method

The data of this study was driven from an ongoing longitudinal population-based survey of the Tehran Lipid and Glucose Study (TLGS), which was implemented in 1999 to investigate the epidemiology of non-communicable diseases risk factors and outcomes on a representative sample of residents of district-13 of Tehran. In this study follow-up visits of participants were performed every three years. Design details of the TLGS have been published previously [31, 32]. This study was carried out in accordance with the declaration of Helsinki guideline. Ethics approval was obtained from the Research Institute for Endocrine Sciences, Shahid Beheshti University of Medical Sciences, Tehran, Iran. Written informed consent was obtained from all participants.

The participants of the present study are 487 women with menopausal aged 40–55 years who are classified into three groups according to the status of menopause. Inclusion criteria included being postmenopausal at baseline and having follow up visits’ data on weight and HTN. Exclusion criteria included women who suffered from HTN at baseline, not having reliable data in terms of age at menopause and menopause reason.

### Measurements

Data on demographic, medical history and reproductive history were acquired by trained staff using interviews and validated questionnaires. Laboratory technicians in the laboratory of the study performed laboratory measurements. Anthropometric measurement was also performed by trained staff. Details of measurement of laboratory measurements, including fasting blood glucose (FBS) levels, low-density lipoprotein cholesterol (LDL), high-density lipoprotein cholesterol (HDL), triglyceride (TG), and total cholesterol (TC) have been reported previously [31, 32]. All individuals had their venous blood drawn between the hours of 7:00 and 9:00 AM, 12 to 14 h after they had stopped eating and 2 to 3 h after waking up. All laboratory kits are supplied by Pars Azmon Inc., Iran. Serum glucose levels was measured using enzymatic colorimetric method by glucose oxidase technique. TC and TG were assessed by enzymatic calorimetric tests with cholesterol esterase and cholesterol oxidase and glycerol phosphate oxidase, respectively. And, HDL was assessed after precipitation of the apolipoprotein B containing lipoproteins with phosphotungistic acid. LDL was calculated by Friedewald’s formula [33].

Height and weight were measured at baseline and each visit. The weight of participants minimally clothed without shoes was recorded using digital scales and recorded to the nearest 100 g. Also, we assessed height without shoes in a standing position. Body mass index (BMI) was considered as weight in kilograms divided by height in meters squared.

After 15 min of rest, a qualified physician measured BP two times in a seated position. Systolic blood pressure (SBP) was identified by phase I Korotkoff sound and diastolic blood pressure (DBP) by Korotkoff phase 5. The average of two assessments was considered.

### Definitions

The time point of 1 year before the 12 months of no menstrual bleeding was regarded as the date of menopause [1]. Menopause occurs due to pathologic and non-pathologic conditions. Natural transition of menopause occurs on an average of 51 years, while estrogen deficiency due to pathological conditions can result in early (40–45 years) [2].

Participants were asked to report the physical activity (PA) that they had participated in within the past 12 months. They were also asked to identify the frequency and duration of each PA. Then each activity is weighted based on its intensity; the acquired amount is known as metabolic equivalent (MET). MET expression is scaled by 3.5 ml/min/kg. For all activity levels, obtained MET was multiplied by the time spent at each level. MET-time from each level was added to a total of 24 h MET-time, representing the average daily level of PA. For analytical purpose, levels of PA were considered low (MET < 600 min / wk) [34].

BMI was defined as normal (BMI < 25.0 kg/m^2^), overweight (25.0–30.0 < kg/m^2^), or obese (30.0 > kg/m^2^).

Weight change [35] was calculated based on the :

$$= {{{\rm{Follow - up}}\,{\rm{measurement - Baseline}}\,{\rm{measurement}}} \over {{\rm{Baseline}}\,{\rm{measurement}}}}\, \times 100$$

Weight change percentage was used for categorizing participants:

1. More than 5% weight loss
2. 3–5% weight loss
3. Less than 3% weight change (stable)
4. 3% to + 5% weight gain
5. More than 5% weight gain

In this study, participants with less than 3% weight change were considered as the reference group. Hypertension was defined as SBP ≥ 140 mm Hg or DBP ≥ 90 mm Hg or use of antihypertensive drugs.

### Statistical analysis

The baseline characteristics of participants were described and compared with normal, non-surgical early menopause, and surgical early menopausal age categories. For the continuous variables, we conducted the Kolmogorov–Smirnov normality test. In the case of rejection of normality assumption, median, interquartile range and Kruskal-Wallis test were used to describe and compare variables, respectively. In cases of non-rejection of the normality assumption, mean, standard deviation and independent ANOVA tests were used, respectively. The categorical variables were described as frequencies (%) and were compared by Chi-squared test or Fisher exact test (For tables with sparse cells).

**Fig. 1:**
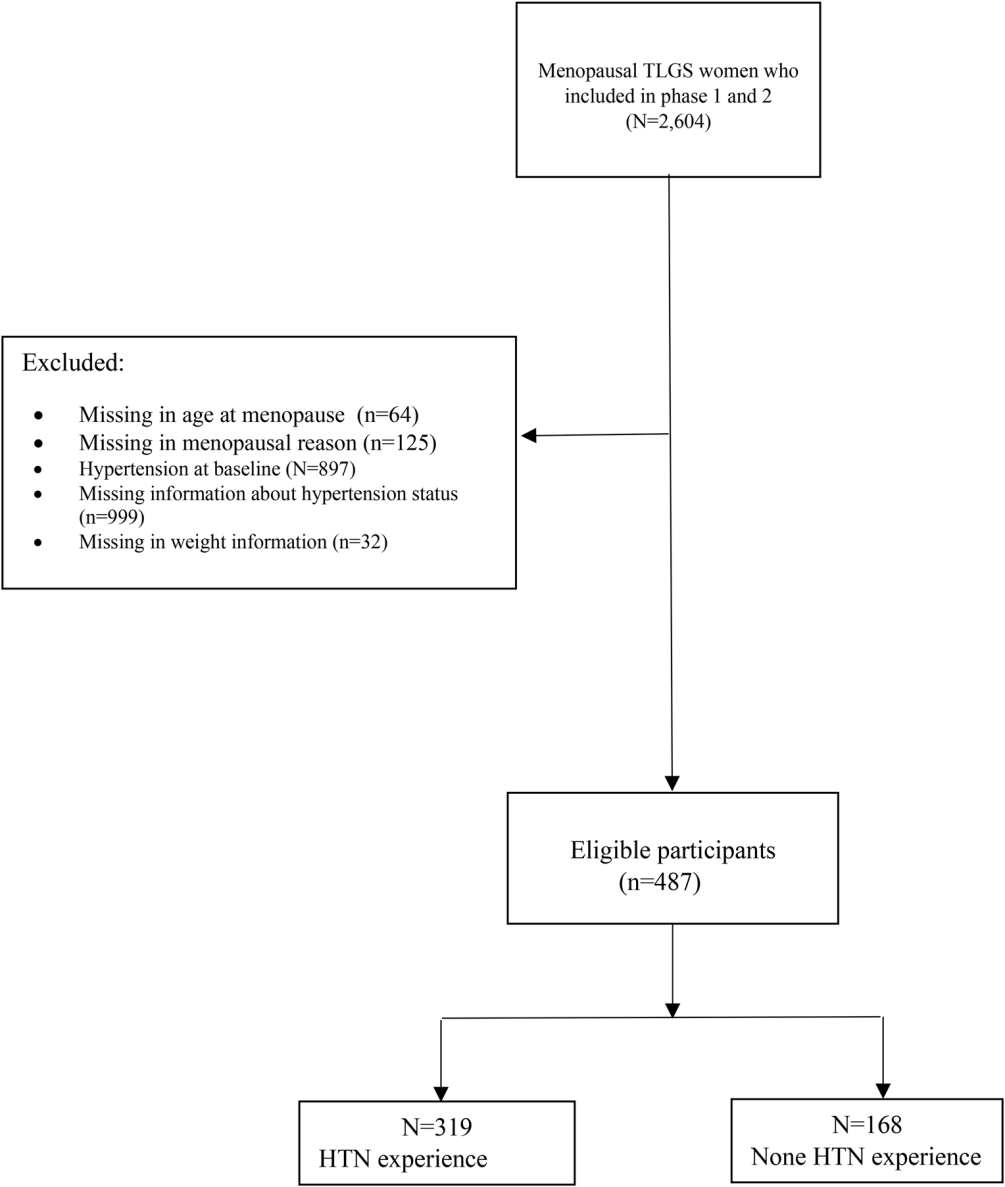
Flowchart of study

Statistical analysis was performed using multivariable Cox proportional hazard regression analysis for modeling and evaluating the association of weight change categories and age of menopausal categories with incidence HTN by reporting hazard ratios with 95% confidence intervals (CI) in two models: model 1: adjusted for age and BMI, model 2: further adjusted for age, BMI, parity, HDL, LDL, TG, TC, FBS, smoking status, physical activity, family history of premature CVD. The time of censoring or outcome of incident hypertension, whichever came first, was considered time to the event. Proportional hazard assumption of Cox models was tested by Z proportional hazard test and plot of the scaled Schoenfeld residuals against time (Supplementary Figure.). Figure 1 shows Kaplan-meier plot for HTN survival based on weight change groups. The mean (SD) of survival time of all participants was 10.51(4.97) years.

We used the ‘survival’ package in the R software version 3.6.0 to fit survival models, including the Cox proportional hazard model. The P-values less than 0.05 were considered statistically significant.

## Results

**Fig. 2:**
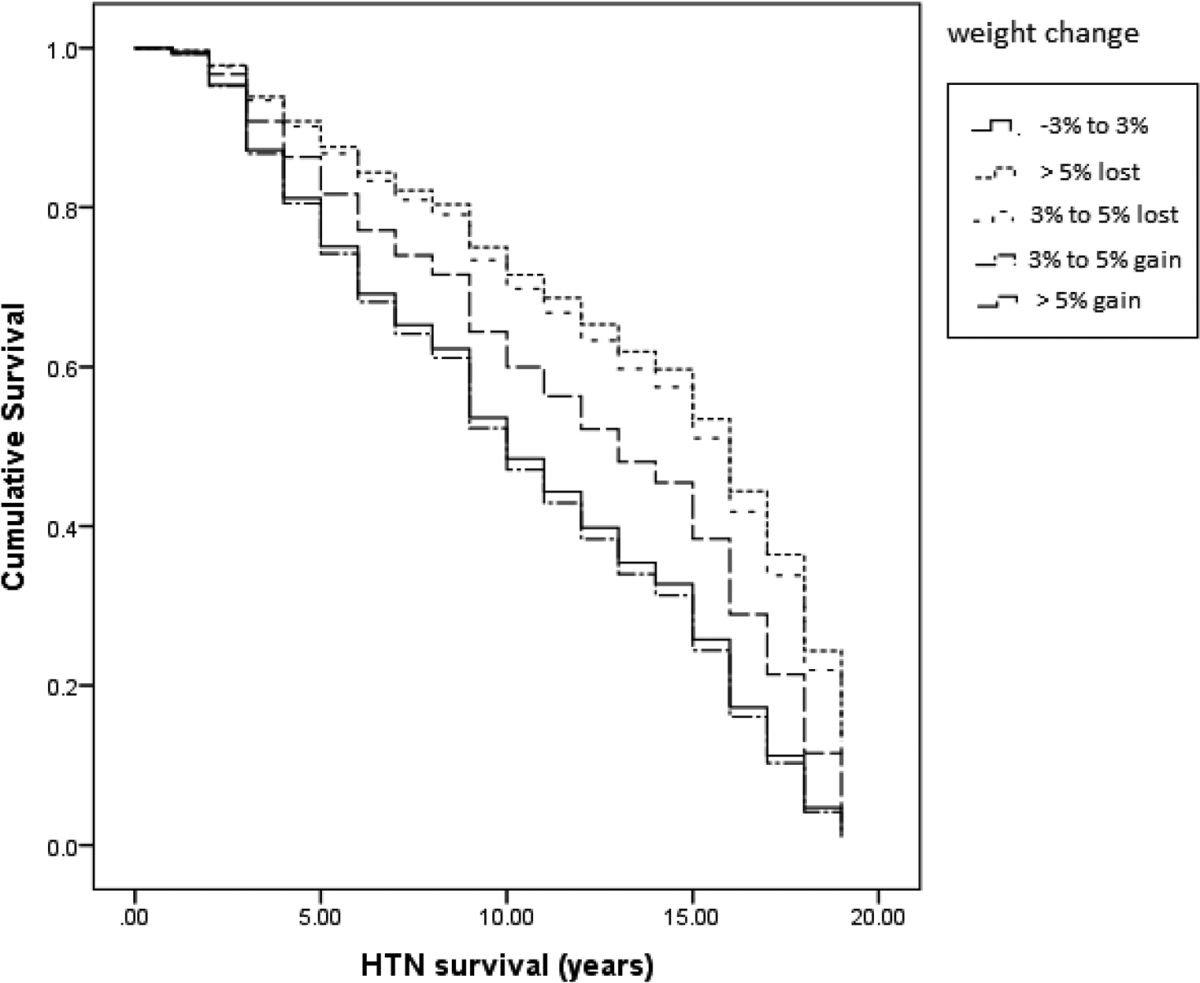
Kaplan-meier plot for HTN survival based on weight changes groups

Four hundred eighty-seven menopausal women by age 56.05 ± 7.37 years old at baseline follow-up participated in this study within the framework of the ongoing Tehran lipid and glucose study. More details of the inclusion and exclusion criteria of participants are presented in Fig. 2.

Table 1 shows the summary of the baseline characteristics of participants and frequencies (%) or median (IQR) for categorical and continuous variables, respectively.

**Table 1:** Characteristics of participants across weight change categories at the baseline and at the last follow up: Tehran Lipid and Glucose Study (TLGS)

Covariates		Weight change categories										Total	
		Lost > 5%		Lost 3–5%		Stable (± 3%)		Gained 3–5%		Gained > 5%			
		(*n* = 113 )		(*n* = 26 )		(*n* = 157 )		(*n* = 47 )		(*n* = 144 )		(*n* = 487 )	
		Baseline	Follow-up	Baseline	Follow-up	Baseline	Follow-up	Baseline	Follow-up	Baseline	Follow-up	Baseline	Follow-up
Continues variables, median(Q1,Q3)													
Age(year), Median (IQR)		56 (52, 62)	71 (67, 76)	55.50 (53.75, 62)	70 (68, 74.25)	56 (51, 61)	70 (65, 75)	57(50, 62)	72.5(66.75, 77.25)	53.50 (50, 59)	68 (65, 73)	55(51, 61)	70 (66, 75)
BMI (kg/m^2^)		29.35 (26.03,31.40)	28.53 (25.01, 30.97)	26.97 (25.83, 29.85)	28.50 (25.73, 30.53)	28.51 (25.88, 31.20)	29.29 (26.63, 32.88)	27.47(25.46, 31.16)	29.38(26.12, 34.04)	27.48 (24.45, 31.19)	30.66 (27.12, 34.75)	28.06 (25.43, 31.20)	29.38(26.4, 32.49)
TG( mg/dl)		172 (124,274.5)	135(98.5, 168)	180.5(100.5, 241.75)	113.5(87.5, 153.65)	156 (117, 229)	124 (99.5, 153.79)	177(125,244)	135(117, 171)	148.5(109.25,203.75)	143(104.6, 171)	160(117, 234)	134.31(101, 164.14)
TC (mg/dl)		249 (204, 277)	194.58 (177, 218.5)	238.5 (231.25, 260)		237(210,272)	193.59(175,213)	242(222, 269)	186(172, 211)	231.5(207, 259.75)	195.17(170.25, 216)	238(209, 269)	193.02(172, 214)
HDL (mg/dl)		43 (35, 53)	51 (46, 60)	46 (35, 53)	54.5 (35, 53)	46 (39, 53)	51.25 (44.76, 58)	42(39, 49)	49 (45, 54)	46(35, 53)	54.5(47.85, 57.88)	46(39, 53)	51(44.52, 57.05)
LDL (mg/dl)		159 (125, 187)	117 (95, 137)	160.6 (125.8, 189)	107.8 (97, 124,41)	155.80 (127.80, 185.50)	114.74 (95.6, 136)	160.6(125.8, 189)	107.8 (97, 124.41)	160.6 (125.8, 189)	107.8(97, 124.41)	153.6(128, 181.8)	113.85(92.8, 135.4)
FBS (mg/dl)		99(89.5, 118.5)	103(90.5, 136.38)	92(88,110.75)	104(91.75, 126.04)	93 (87, 107.5)	98 (89, 119)	94(88, 103)	97(88,109)	92(86, 101.75)	97(90,114.75)	94(87, 107)	98(90, 119)
Parity (number)		5 (3, 6)	-	4(3, 6)	-	5 (3, 6)	-	4 (3, 7)	-	4 (3, 6)	-	5 (3, 6)	-
Categorical variables, number (%)													
Menopausal age	Normal	71 (62.4)	-	23 (88.5)	-	98 (62.4)	-	33 (70.2)	-	80 (55.6)	-	305(62.6)	-
	Early	19 (16.8)	-	1 (3.8)	-	26 (16.6)	-	4 (8.5)	-	25 (17.4)	-	75(15.4)	-
	early Surgical	23 (20.4)	-	2 (7.7)	-	33 (21)	-	10 (21.3)	-	39 (27.1)	-	107(22)	-
Smoking status (Yes)		10 (8.8)	8 (8.6)	0 (0)	1 (5)	8 (5.1)	6 (4.8)	4 (8.5)	3 (7.9)	8 (5.6)	4 (3.2)	30 (6.2)	22 (5.5)
Physical activity (appropriate) number (%)		43 (38.1)	34 (30.1)	7 (26.9)	9 (34.6)	41 (26.1)	43 (27.4)	17 (36.2)	7 (14.9)	46 (31.9)	36 (25)	154(31.6)	129 (26.5)
History of family premature CVD (Yes)		12 (10.6)	-	5 (19.2)	-	19 (12.1)	-	10 (21.3)	-	30 (20.8)	-	76(15.6)	-
Incident of HTN (Yes)		-	58 (51.3)	-	14 (53.8)	-	115 (73.2)	-	37 (78.7)	-	95 (66)	-	319(65.5)
Survival time HTN		-	13 (9, 15)	-	12.5 (9, 16)	-	9 (5, 15)	-	9 (4, 15)	-	12 (7, 16)	-	11 (6,15)

*Abbreviations* CVD: Cardiovascular disease; HTN: hypertension; BMI: body mass index; FBS: fasting blood glucose; TG: triglyceride; LDL: low-density lipoprotein cholesterol; HDL: high-density lipoprotein cholesterol; TC: total cholesterol

Most subjects in the natural and early-surgical groups were overweight at baseline; however, most nonsurgical early menopause subjects were obese. Also, in the last follow-up, the majority of all groups were obese. However, the group’s baseline and last follow-up differences were insignificant. Table 2 shows the comparison of BMI categories in baseline and follow-up of the study based on menopausal categories (natural, non-surgical and surgical early menopause).

**Table 2:** Comparison of BMI categories in baseline and follow up of study based on menopausal age categories

BMI	Menopausal age					
	Baseline			Last follow up		
	Normal	Early-nonsurgical	Early-surgical	Normal	Early-nonsurgical	Early-surgical
Normal	64(21)	20 (26.7)	18(16.8)	58(19)	13(17.3)	9 (8.4)
Overweight	140(45.9)	23(30.7)	49(45.8)	121(39.7)	27(36)	41(38.3)
Obese	101(33.1)	32(42.7)	40(37.4)	126(41.3)	35(46.7)	57(53.3)
P-value	0.13			0.08		

*Abbreviations* BMI: body mass index

Results of log rank test to evaluate significance of difference between survivals in weigh changes groups was:

$$Chisq = {\rm{ }}23.1\,{\rm{ }}on\,{\rm{ }}4\,\,degrees{\rm{ }}\,of\,{\rm{ }}freedom,{\rm{ }}p < 0.001$$

Compared to those with stable weight, women with weight loss of > 5% and 3–5% had age- and BMI-adjusted HRs of 0.50 (CI 95%, 0.36, 0.69; *p* < 0.001) and 0.49 (CI 95%, 0.28, 0.86; *p* = 0.01), respectively. Furthermore, for incident HTN, FBS (HR: 1.01, CI 95%:1.00, 1.01; *p* < 0.001) and smoking (HR: 1.66, CI 95%; 1.01, 2.50; *p* = 0.015) were positively associated with HTN. After controlling adjusted for age, BMI, parity, HDL, LDL, TG, TC, FBS, smoking status, physical activity, family history of premature CVD in model 2, the association between weight loss of > 5%, weight loss 3–5%, FBS, smoking and HTN still remained significant. Also, other factors including weight gain > 5% HRs of 0.69 (CI 95%, 0.51, 0.91; *p* = 0.01), age (HR: 1.04; CI 95%, 1.01, 1.08, *p* = 0.004), BMI (HRs: 1.02, CI 95%, 1.00, 1.05; *p* = 0.03) were significantly associated with HTN. (Table 3)

**Table 3:** The multivariable hazard ratio (HR) and 95% confidence intervals (CI) of association between weight change categories and incident HTN: Tehran Lipid and Glucose Study (TLGS), Iran, 1999–2018

Covariates		Model 1^*^			Model 2^**^		
		HR (95% CI)	P-value	P-value***	HR (95% CI)	P-value	P-value***
Weight change categories ref = ± 3% (normal)	Lost > 5%	0.50(0.36, 0.69)	< 0.001	0.26	0.44 (0.32,0.62)	< 0.001	0.39
	Lost 3–5%	0.49(0.28, 0.86)	0.01		0.47 (0.26,0.84)	0.01	
	Gained 3–5%	1.10(0.76, 1.61)	0.58		1.02 (0.70,1.50)	0.88	
	Gained > 5%	0.76(0.57, 1.00)	0.05		0.69 (0.51,0.91)	0.01	
Menopausal age (ref = Normal)	Early	0.73(0.52, 1.02)	0.07	0.79	0.83 (0.56,1.23)	0.36	
	Surgical early	0.96(0.70, 1.32)	084		1.03 (0.68,1.54)	0.88	
Difference of menopause age and baseline age(ref = 0–5 years)	5–10 years	0.95(0.70, 1.30)	0.78	0.81	0.93 (0.67,1.31)	0.71	
	> 10 years	0.89(0.64, 1.25)	0.52		0.91 (0.58,1.42)	0.69	
Age (Years)	-	-	-	-	1.04 (1.01,1.08)	0.004	
BMI (Kg/m^2^)	-	-	-	-	1.02 (1.00,1.05)	0.030	
Parity	-	1.04(0.99, 1.10)	0.06	0.77	1.04 (0.98,1.09)	0.13	
HDL	-	0.99(0.98, 1.00)	0.09	0.75	0.99 (0.97,1.01)	0.60	
LDL	-	0.99(0.99, 1.00)	0.75	0.58	0.99 (0.98,1.01)	0.84	
TG	-	1.00(0.99, 1.00)	0.47	0.85	1.00 (0.99,1.00)	0.85	
FBS	-	1.01(1.00, 1.01)	< 0.001	0.71	1.00 (1.00,1.01)	< 0.001	
TC	-	0.99(0.99, 1.00)	0.70	0.55	0.99 (0.98,1.01)	0.97	
Smoking status (ref = No)	Yes	1.66 (1.010,2.50)	0.015	0.37	1.70 (1.11,2.58)	0.01	
Physical activity (ref = Low)	Appropriate	0.88(0.69, 1.13)	0.33	0.38	0.97 (0.75,1.24)	0.82	
Family history of CVD (ref = No)	Yes	1.31(0.98, 1.74)	0.06	0.87	1.23 (0.90,1.68)	0.18	

* model 1:first adjusted Cox model (adjusted for age and BMI)

** model 2:further adjusted Cox model (adjusted for age, BMI, parity, HDL, LDL, TG,TC, FBS, smoking status, physical activity, family history of CVD)

*** P-value of Z proportionality hazard test for Cox models

*Abbreviations* CVD: Cardiovascular disease; HTN: hypertension; BMI: body mass index; FBS: fasting blood glucose; TG: triglyceride; LDL: low-density lipoprotein cholesterol; HDL: high-density lipoprotein cholesterol; TC: total cholesterol

## Discussion

The result of this longitudinal population-based study showed that weight loss (3–5% and > 5%) and weight gain (> 5%) had reduced the risk of HTN, while there was no discernible change in the incidence of HTN among women with stable weight change and 3–5% weight gain. Furthermore, baseline age, BMI, FBS and smoking were significantly related to incident HTN.

Our result showed that the risk of HTN decreased among women with 5% and 3–5% weight loss and > 5% weight gain. Several studies have highlighted the role of weight in the development of HTN. Williams (2008), in a study among 4,550 men and 10,111 women who followed for an average of seven years, found that for those who gained ≥ 2.4kg/m^2^, almost 70% were more prone to HTN [36]. A 14-year follow-up study among 2,560 men and women showed that 41.95% of participants maintained a normal weight, 33.98% maintained an overweight/obese status, 18.83% became overweight/obese, and 5.24% had lower BMI. Among different categories of weight change, participants who were weight-gainers and normal weight-maintainers were more prone to HTN than others [37]. In our study, the majority of women were obese/overweight at baseline and weight loss led to improvement of BP among them over time. The obesity paradox can also explain the negative impact of weight gain on HTN risk among this study’ participants. It is proposed that overweight or obesity in the elderly can lead to production of adipokine by adipose as a cardioprotective [38]. Recent study highlighted that pro-rich inequality affects the prevalence and control of HTN in Iran [39]. It seems that the implication of measuring the weight changes in order to better manage HTN among postmenopausal women is essential.

A recent meta-analysis showed that each 1-kilogram weight loss could result in a declining 1 mm Hg in systolic and diastolic blood pressure [40]. It is also documented that some studies have shown a paradoxical association between weight change and mortality rate; in other words, weight loss was associated with an increased mortality rate; by contrast, the mortality rate decreased among people who had weight gain [41]. There are inconsistencies across studies. A meta-analyze reported that weight loss could decrease the risk of HTN by 24–40% and 40–54% among overweight and obese participants, respectively [42]. In our study, there was significant association between one category of weight gain (> 5%) and the decreased risk of HTN. However, Underland et al. (2022) in their study among 5,039 women with average age of 78.76 ± 6.92 found that weight gain was not associated with mortality or cardiovascular outcomes, however weight loss of 5% or more was associated with increased risk of mortality [43]. Women who experienced the surgical menopause might be at increased risk of coronary heart disease than women with natural menopause [7]. The decreased risk of HTN in cases with weight loss can be attributed to the favorable improvement in metabolic parameters. The lipid risk profile of all participants in the last follow-up was better than the baseline. However, the FBS profile at the last follow-up was worsened. It is possible that unmeasured factors could be the root cause of changes in risk factors as well as the cause of weight change. Overall, there are some methodological differences and population characteristics between our study and the mentioned studies, which may have contributed to differences in results. Nevertheless, further studies are needed to clarify the association between weight change and the risk of hypertension after menopause.

Previous studies addressing the association between HTN and weight change among postmenopausal women are relatively scarce. Results from the Weight Loss Maintenance Trial showed that in participants whose weight increased > 3% with mean age 55 years, SBP increased significantly at 60 months [44]. Moazzeni et al. (2022) also reported that weight gain > 5% was associated with better cardiovascular outcomes among people with diabetes [45]. In the Trials of Hypertension Prevention, among individuals 30-54 years with high normal BP, weight change was associated with mortality [46]. Study of Li et al., (2022) showed that age onset of obesity at ≥ 60 years of age was not associated with risk of HTN [47]. In this study, we have no information of age onset of overweight or obesity of participants.

We also found that age, FBS levels, smoking and BMI are factors that contribute to increasing the risk of HTN. So far, the literature determined the traditional risk factors of HTN including general adiposity, abdominal adiposity, alcohol consumption, smoking, diet, and physical inactivity [48]. Age is considered a powerful predictor of HTN [49]. Aging process among women due to the differences in gene expression, alteration of sex hormones, and function of the cardiovascular system can differently affect the HTN risk among women compared to the men [50]. It is proposed that the association between biologic aging and HTN is mainly related to the underlying mechanisms including inflammation, oxidative stress, endothelial dysfunction, and neurohumoral dysfunction [51, 52]. In the Women’s Health Study also smoker women aged ≥ 45 years old were modestly at higher risk of HTN [53]. Kin et al. (2019) revealed that female smokers older than 29 years old were more prone to experience HTN [54]. With regard to FBS, a study among middle-aged Japanese people, FBS regardless of insulin resistance was associated with higher risk of HTN [55]. With an aging population, awareness of Iranian people regarding the risk factors of non-communicable diseases’ risk factors is not sufficient, so policymakers should develop national strategies to improve prevention, detection, and management of HTN in Iran [56].

It is unclear how weight changes in postmenopausal women can affect the risk of developing HTN. Different mechanisms clarify the association between weight change and the risk of HTN. Firstly, estrogen depletion could result in endothelial dysfunction and weight gain, leading to increased oxidative stress, renal vasoconstriction, and hypertension [22, 57]. Furthermore, estrogen deficiency, in different ways, such as energy regulation, controls the central nucleus of appetite and satiety and the secretion of adipokine, which are obesity triggers [58]. To sum up, the main factors which contributed to the development of HTN include sex steroids (rise of estrogen, drop of testosterone), environmental factors (age, BMI, insulin oxidative stress, cholesterol) and gens ( renin-angiotensin system, adrenergic, eNOS, estrogen-related aromatase) [22]. Hence, combining these factors could play a role in HTN. Obesity-related hypertension develops due to the overactivation of the sympathetic nervous system, stimulation of the renin-angiotensin-aldosterone system, alterations in adipose-derived cytokines such as leptin, and insulin resistance [59]. It is hypothesized that the early onset of menopause (surgical, non-surgical) due to the absence of the protective effect of estrogens would result in a higher risk of HTN in women rather than natural menopause. However, in our study, we could not find this association. Wu et al. (2023) reported that women experiencing menopause at a late age were at risk of obesity-mediated HTN [60]. Also, a cross-sectional study of 3,406 postmenopausal women reported that earlier menopausal age was associated with hypertension [61]. The late menopausal transition is also associated with hypertension [62]. It should be noted that apart from the effect of menopause on blood pressure, other factors like ageing could mask the menopausal effect on blood pressure.

There are some limitations and strengths of this study which should be considered in the interpretation of findings. A number of strengths of this study include the large sample size, measurement based on the standard protocol of TLGS and a valid and reliable questionnaire. In this study, unlike other studies, the weight measurement was not self-reported in our study. In this cohort studies loss to follow-up represents might lead to the selection bias, there was no statistically significant difference in background variables between those lost to follow up and those included in the present study (data has not shown). For assessment of physical activity, we used a questionnaire, which may increase recall bias.

In this study, the baseline measurement of data entry to study did not coincide with the exact time of menopause; weight change which occurred during the interval between menopause and the first phase of TLGS was not considered in this study. Although adjustment for measured confounders was performed in this study, other unmeasured confounders, such as lifestyle, genetics, etc., might affect the observed findings. The sample size for groups whose weight change was ± 3–5% was small. The cause of unwanted weight change was not evaluated in this study. The menopausal age in this study was a self-reported variable, so recall bias should be considered. The possible misclassification in the type of menopause due to the recall bias should be noticed. While our study population was from the urban area, which limits the generalizability to the rural area.

## Conclusion

In this study, weight loss and gain have a protective impact on the development of HTN. However, more evidence is needed to confirm this association. Our study supports the importance of weight change in clinical practice to evaluate HTN. Through counseling, clinicians can help menopausal women to monitor their weight change.

### Electronic supplementary material

Below is the link to the electronic supplementary material.

**Supplementary Material 1: Supplementary Figure**. Plot of the scaled Schoenfeld residuals against time for variables in adjusted model

## Data Availability

The datasets used and/or analyzed during the current study available from the corresponding author on reasonable request.

## Declarations

All methods were carried out in accordance with relevant guidelines and regulations with the Declaration of Helsinki.

## Abbreviations

CVD: cardiovascular disease
HTN: Hypertension
BMI: Body mass index
MET: metabolic equivalent
PA: Physical activity
TLGS: Tehran lipid and glucose
CI: confidence intervals
HR: hazard ratio
FBS: fasting blood sugar
LDL: low-density lipoprotein cholesterol
HDL: high-density lipoprotein cholesterol
TC: total cholesterol
ESC/ESH: European Society of Cardiology and European Society of Hypertension
BP: blood pressure
DASH: Low-sodium Dietary Approaches to Stop Hypertension
PA: physical activity

## References

[1] Endocrinology TLD. Menopause: a turning point for women’s health. : S2213-8587 (2222) 00142–5.

[2] Shifren JL, Gass ML (2014). The North American Menopause Society recommendations for clinical care of midlife women. Menopause.

[3] Gold EB (2011). The timing of the age at which natural menopause occurs. Obstet Gynecol Clin North Am.

[4] Lovejoy JC (1998). The influence of sex hormones on obesity across the female life span. J Womens Health.

[5] Shifren JL, Gass ML, Group NRfCCoMWW (2014). The North American Menopause Society recommendations for clinical care of midlife women. Menopause.

[6] Rosano G, Vitale C, Marazzi G, Volterrani M (2007). Menopause and cardiovascular disease: the evidence. Climacteric.

[7] El Khoudary SR, Aggarwal B, Beckie TM, Hodis HN, Johnson AE, Langer RD, Limacher MC, Manson JE, Stefanick ML, Allison MA (2020). Menopause transition and cardiovascular disease risk: implications for timing of early prevention: a scientific statement from the American Heart Association. Circulation.

[8] Graff-Iversen S, Thelle DS, Hammar N (2008). Serum lipids, blood pressure and body weight around the age of the menopause. Eur J Cardiovasc Prev Rehabil.

[9] Karvonen-Gutierrez C, Kim C. Association of mid-life changes in body size, body composition and obesity status with the menopausal transition. In: *Healthcare: 2016*: MDPI; 2016: 42. 10.3390/healthcare4030042 PMC5041043 27417630

[10] Davis SR, Castelo-Branco C, Chedraui P, Lumsden M, Nappi R, Shah D, Villaseca P (2012). Day WGotIMSfWM: understanding weight gain at menopause. Climacteric.

[11] Murphy E (2011). Estrogen signaling and cardiovascular disease. Circ Res.

[12] Ashraf MS, Vongpatanasin W (2006). Estrogen and hypertension. Curr Hypertens Rep.

[13] Rurik I, Móczár C, Buono N, Frese T, Kolesnyk P, Mahlmeister J, Petrazzuoli F, Pirrotta E, Ungvari T, Vaverkova I (2017). Early and menopausal weight gain and its relationship with the development of diabetes and hypertension. Exp Clin Endocrinol Diabetes.

[14] Hruby A, Hu FB (2015). The epidemiology of obesity: a big picture. PharmacoEconomics.

[15] Mittal BV, Singh AK (2010). Hypertension in the developing world: challenges and opportunities. Am J Kidney Dis.

[16] Rappelli A (2002). Hypertension and obesity after the menopause. J Hypertens Suppl.

[17] Antza C, Doundoulakis I, Stabouli S, Kotsis V (2021). American, European and international hypertension guidelines: time to shake hands?. Int J Cardiol Hypertens.

[18] Eghbali-Babadi M, Khosravi A, Feizi A, Alikhasi H, Kheirollahi N, Sarrafzadegan N (2021). Prevalence of pre-hypertension and hypertension, awareness, treatment, and control of hypertension, and cardiovascular risk factors in postmenopausal women. ARYA Atheroscler.

[19] Zamandi M, Daroudi R, Sari AA (2023). Direct costs of Hypertension Treatment in Iran. Iran J Public Health.

[20] Barton M, Meyer MR (2009). Postmenopausal hypertension: mechanisms and therapy. Hypertension.

[21] Bagdey PS, Ansari JA, Barnwal RK (2019). Prevalence and epidemiological factors associated with hypertension among post-menopausal women in an urban area of central India. Clin Epidemiol Global Health.

[22] Coylewright M, Reckelhoff JF, Ouyang P (2008). Menopause and hypertension: an age-old debate. Hypertension.

[23] Nazarpour S, Simbar M, Tehrani FR (2016). Factors affecting sexual function in menopause: a review article. Taiwan J Obstet Gynecol.

[24] Kabodi S, Ajami E, Zakiei A, Zangeneh A, Saeidi S (2019). Women’s quality of life in menopause with a focus on hypertension. J Obstet Gynecol India.

[25] Loaiza-Betancur AF, Chulvi-Medrano I, Díaz-López VA, Gómez-Tomás C (2021). The effect of exercise training on blood pressure in menopause and postmenopausal women: a systematic review of randomized controlled trials. Maturitas.

[26] Bertochi GFA, Oliveira RFd S, IAd B, Neto O, Sasaki JE. Sedentary postmenopausal women not undergoing hormone replacement therapy can have their blood pressure lowered by performing resistance training: a systematic review and meta-analysis of randomized controlled trials. Motriz: Revista De Educação Física 2022, 28.

[27] Nowson CA, Wattanapenpaiboon N, Pachett A (2009). Low-sodium Dietary approaches to stop hypertension-type diet including lean red meat lowers blood pressure in postmenopausal women. Nutr Res.

[28] Ko S-H, Kim H-S (2020). Menopause-Associated lipid metabolic disorders and foods Beneficial for Postmenopausal Women. Nutrients.

[29] Lizcano F, Guzmán G (2014). Estrogen Deficiency and the origin of obesity during menopause. Biomed Res Int.

[30] Iorga A, Cunningham CM, Moazeni S, Ruffenach G, Umar S, Eghbali M (2017). The protective role of estrogen and estrogen receptors in cardiovascular disease and the controversial use of estrogen therapy. Biol Sex Differ.

[31] Azizi F, Ghanbarian A, Momenan AA, Hadaegh F, Mirmiran P, Hedayati M, Mehrabi Y, Zahedi-Asl S (2009). Prevention of non-communicable disease in a population in nutrition transition: Tehran lipid and glucose study phase II. Trials.

[32] Azizi F, Zadeh-Vakili A, Takyar M (2018). Review of Rationale, Design, and initial findings: Tehran lipid and glucose study. Int J Endocrinol Metabolism.

[33] Azizi F, Ghanbarian A, Momenan AA, Hadaegh F, Mirmiran P, Hedayati M, Mehrabi Y, Zahedi-Asl S, Lipid T (2009). Ir GSGaea: Prevention of non-communicable disease in a population in nutrition transition: Tehran lipid and glucose study phase II. Trials.

[34] Momenan AA, Delshad M, Sarbazi N, Rezaei Ghaleh N, Ghanbarian A, Azizi F. Reliability and validity of the Modifiable Activity Questionnaire (MAQ) in an Iranian urban adult population. Arch Iran Med. 2012;15(5):279–82. PMID: 22519376. 22519376

[35] Stevens J, Truesdale KP, McClain JE, Cai J (2006). The definition of weight maintenance. Int J Obes (Lond).

[36] Williams PT (2008). Increases in weight and body size increase the odds for hypertension during 7 years of follow-up. Obes (Silver Spring).

[37] Mahwati Y. Effect of body weight changes on hypertension in Indonesian adults (a 14-year follow up). 2019.

[38] Donini LM, Pinto A, Giusti AM, Lenzi A, Poggiogalle E (2020). Obesity or BMI paradox? Beneath the tip of the iceberg. Front Nutr.

[39] Mahdavi M, Parsaeian M, Farzadfar F, Mohamadi E, Olyaeemanesh A, Takian A (2022). Inequality in prevalence, awareness, treatment, and control of hypertension in Iran: the analysis of national households’ data. BMC Public Health.

[40] Neter JE, Stam BE, Kok FJ, Grobbee DE, Geleijnse JM (2003). Influence of weight reduction on blood pressure: a meta-analysis of randomized controlled trials. Hypertension.

[41] Myers J, Lata K, Chowdhury S, McAuley P, Jain N, Froelicher V (2011). The obesity paradox and weight loss. Am J Med.

[42] Poorolajal J, Hooshmand E, Bahrami M, Ameri P (2017). How much excess weight loss can reduce the risk of hypertension?. J Public Health (Oxf).

[43] Underland LJ, Schnatz PF, Wild RA, Saquib N, Shadyab AH, Allison M, Banack H, Wassertheil-Smoller S (2022). The impact of weight change and measures of physical functioning on mortality. J Am Geriatr Soc.

[44] Tyson CC, Appel LJ, Vollmer WM, Jerome GJ, Brantley PJ, Hollis JF, Stevens VJ, Ard JD, Patel UD, Svetkey LP (2013). Impact of 5-year weight change on blood pressure: results from the weight loss maintenance trial. J Clin Hypertens (Greenwich).

[45] Moazzeni SS, Hizomi Arani R, Deravi N, Hasheminia M, Khalili D, Azizi F, Hadaegh F (2021). Weight change and risk of cardiovascular disease among adults with type 2 diabetes: more than 14 years of follow-up in the Tehran lipid and glucose study. Cardiovasc Diabetol.

[46] Cook NR, Appel LJ, Whelton PK (2018). Weight change and mortality: long-term results from the trials of hypertension prevention. J Clin Hypertens (Greenwich).

[47] Li W, Fang W, Huang Z, Wang X, Cai Z, Chen G, Wu W, Chen Z, Wu S. Association between age at onset of overweight and risk of hypertension across adulthood. 2022, 108(9):683–8. 10.1136/heartjnl-2021-320278 PMC8995813 35190372

[48] Andriolo V, Dietrich S, Knüppel S, Bernigau W, Boeing H (2019). Traditional risk factors for essential hypertension: analysis of their specific combinations in the EPIC-Potsdam cohort. Sci Rep.

[49] Hustrini NM, Susalit E, Rotmans JI (2023). 61. Older Age is the strongest risk factors for hypertension in Indonesia: Subgroup Analysis of the National Basic Health Survey 2018. J Hypertens.

[50] Kim KI (2022). Hypertension in older women: the biggest challenge for Cardiovascular Health in an aged society. Korean Circ J.

[51] Buford TW (2016). Hypertension and aging. Ageing Res Rev.

[52] Sun Z (2015). Aging, arterial stiffness, and hypertension. Hypertension.

[53] Bowman TS, Gaziano JM, Buring JE, Sesso HD (2007). A prospective study of cigarette smoking and risk of incident hypertension in women. J Am Coll Cardiol.

[54] Kim SH, Lee JS. The association of smoking and hypertension according to cotinine-verified smoking status in 25,150 Korean adults. 2019, 41(5):401–8. 10.1080/10641963.2018.1489548 30059635

[55] Tatsumi Y, Asayama K, Morimoto A, Sonoda N, Miyamatsu N, Ohno Y, Miyamoto Y, Izawa S, Ohkubo T, HIGH FASTING BLOOD GLUCOSE LEVEL INCREASE RISK OF HYPERTENSION INCIDENCE INDEPENDENT OF INSULIN RESISTANCE IN JAPANESE (2021). THE SAKU STUDY. J Hypertens.

[56] Azadnajafabad S, Mohammadi E, Aminorroaya A, Fattahi N, Rezaei S, Haghshenas R, Rezaei N, Naderimagham S, Larijani B, Farzadfar F. Non-communicable diseases’ risk factors in Iran; a review of the present status and action plans. J Diabetes Metabolic Disorders 2021:1–9. 10.1007/s40200-020-00709-8 PMC7821170 33500879

[57] Dubnov G, Brzezinski A, Berry EM (2003). Weight control and the management of obesity after menopause: the role of physical activity. Maturitas.

[58] Lizcano F, Guzmán G (2014). Estrogen Deficiency and the origin of obesity during menopause. Biomed Res Int.

[59] Shariq OA, McKenzie TJ (2020). Obesity-related hypertension: a review of pathophysiology, management, and the role of metabolic surgery. Gland Surg.

[60] Wu Y-J, Jiang C-Q, Zhu T, Jin Y-L, Zhu F, Zhou B-J, Xu L, Zhang W-S (2023). Obesity indicators as mediators of the association between age at menopause and blood pressure values. Hypertens Res.

[61] Song L, Shen L, Li H, Liu B, Zheng X, Zhang L, Liang Y, Yuan J, Wang Y (2018). Age at natural menopause and hypertension among middle-aged and older Chinese women. J Hypertens.

[62] Son MK, Lim N-K, Lim J-Y, Cho J, Chang Y, Ryu S, Cho M-C, Park H-Y (2015). Difference in blood pressure between early and late menopausal transition was significant in healthy Korean women. BMC Womens Health.

