# Supplementary material for: To what extent the weight changes impact the risk of hypertension among menopausal women: insights from Tehran lipid and glucose study
Source: BMC Womens Health. 2024 Feb 19;24:128. doi: 10.1186/s12905-024-02974-8 (PMC10877883; doi:10.1186/s12905-024-02974-8)
Supplement: Supplementary file 1 — Supplementary Material 1: Supplementary Figure. Plot of the scaled Schoenfeld residuals against time for variables in adjusted model [file 12905_2024_2974_MOESM1_ESM.docx]

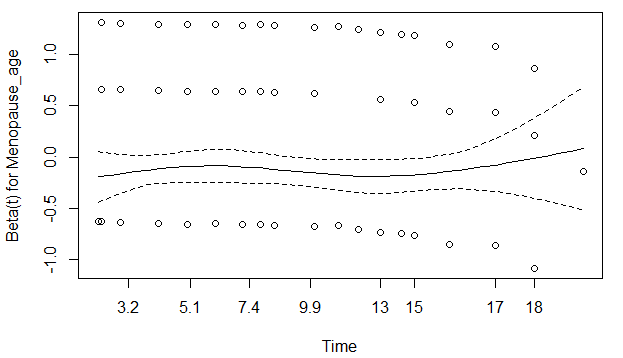

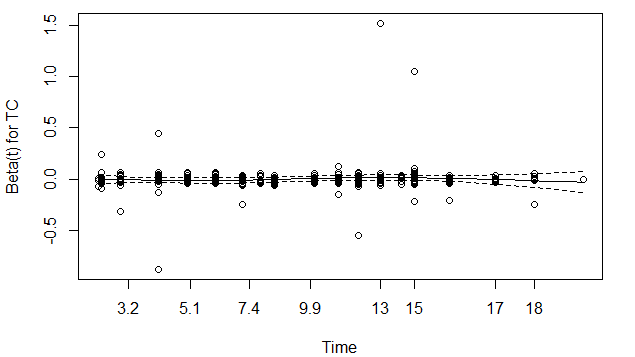


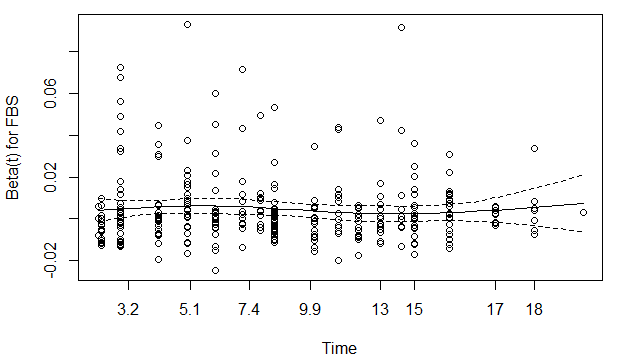

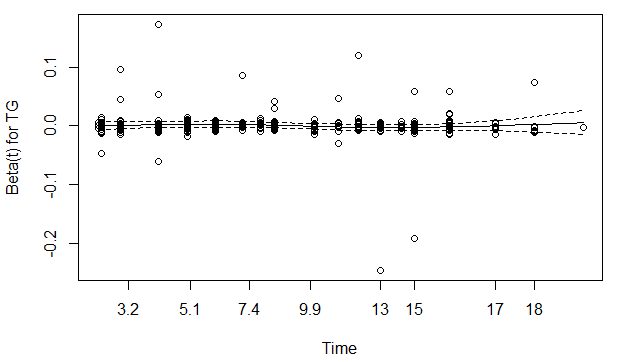


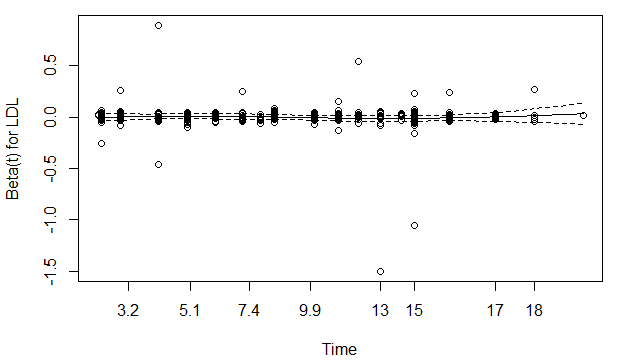

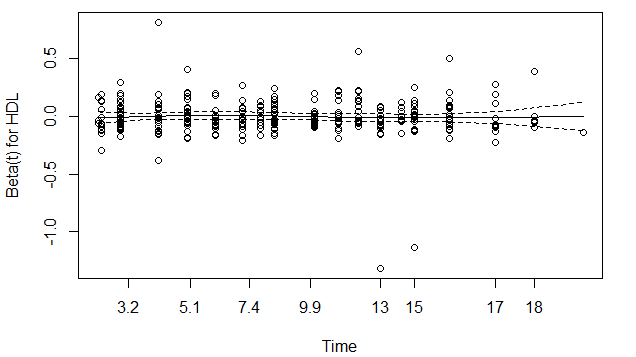


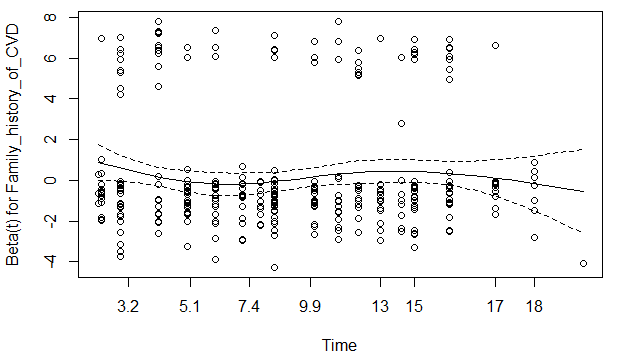

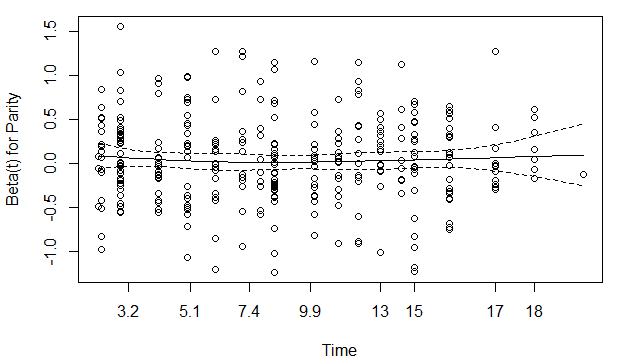


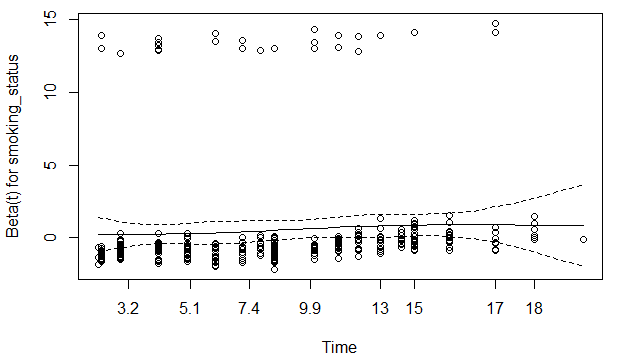

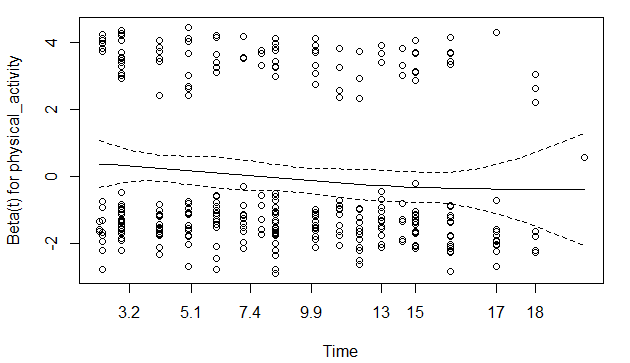


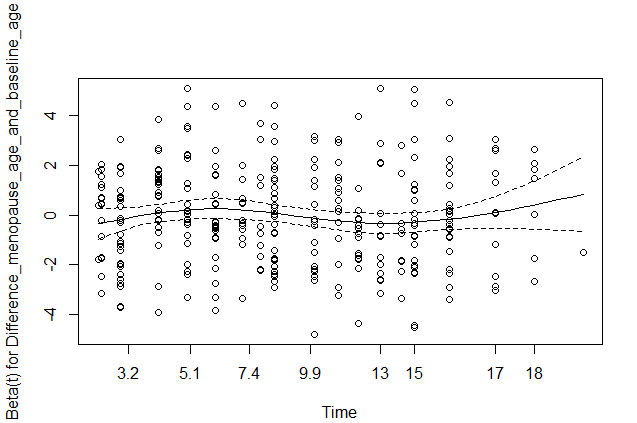

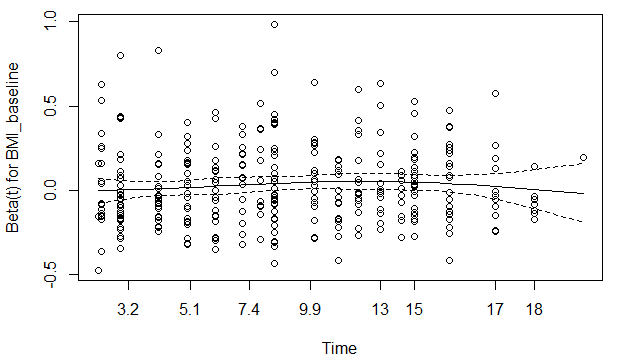


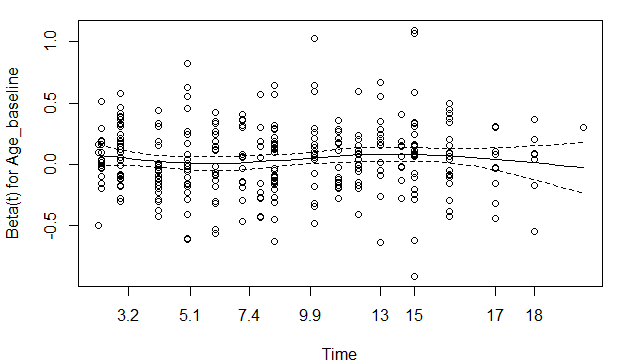


Supplementary Figure. Plot of the scaled Schoenfeld residuals against time for variables in adjusted model
